# Exploring early-stage orienting behavior using an eye tracker for attention deficit hyperactivity disorder classification

**DOI:** 10.1038/s41598-026-41419-0

**Published:** 2026-02-26

**Authors:** Seonmi Lee, Sangil Lee, Inji Jeong, Jaehyun Jeong, Hyoju Park, Mee-Kyoung Kwon, Theodore Zanto, Sunhae Sul, Dooyoung Jung

**Affiliations:** 1 Graduate School of Health Science and Technology, Ulsan National Institute of Science and Technology, Ulsan, 44919 Republic of Korea; 2 School of Liberal Arts, Ulsan National Institute of Science and Technology, Ulsan, 44919, Republic of Korea; 3 LVIS Korea, Daegu, 42710 Republic of Korea; 4 Department of Psychology, Pusan National University, Pusan, 46241 Republic of Korea; 5 Department of Child Studies, Seoul Women’s University, Seoul, 01797 Republic of Korea; 6 Neuroscape, Department of Neurology, University of California, San Francisco, CA 94158 USA; 7 School of Digital Humanities and Computational Social Sciences, Korea Advanced Institute of Science and Technology, Daejeon, 34141 Republic of Korea; 8 Department of Data Science, Hanyang University, Seoul, 04763 Republic of Korea

**Keywords:** Early-stage orienting behavior, ADHD, Eye movement, Attention shifts, Classification, Gaze-cueing task, Diseases, Neuroscience, Psychology, Psychology

## Abstract

Exploring early-stage orienting behavior is essential for elucidating the behavioral mechanisms underlying attentional shifts in attention deficit hyperactivity disorder (ADHD). However, traditional tasks lacking eye-tracking data often obscure these mechanisms. This study investigates low-level attentional shifting in ADHD using a simplified gaze-cueing task and explores classification markers via eye movement. Eye-tracking data were analyzed from 27 typically developing children and 19 children diagnosed with ADHD. We constructed a logistic regression model for classification purposes. Eye movement data alone yielded an accuracy of 0.84, comparable to the accuracy achieved using combined eye-tracking and behavioral data (0.87), underscoring the sensitivity of gaze-based features. Children with ADHD exhibited significantly prolonged inter-saccadic fixations in non-target regions (*p* = .02, d = 0.80) and marginally reduced saccade frequency (*p* = .06, d = − 0.52) during target detection, indicating delayed attentional shifting and diminished goal-directed attention. Prolonged fixation during target detection behavior emerged as the strongest predictor, correlating with both inattention and hyperactivity (*r* = .46; *r* = .36; both *p* < .01). Additionally, children with ADHD demonstrated lower response to joint attention and a greater reliance on peripheral vision. These findings highlight distinct gaze patterns under low cognitive load, revealing subtle mechanisms of executive dysfunction and potential early classification markers.

**Supplementary Information:**

The online version contains supplementary material available at 10.1038/s41598-026-41419-0.

## Introduction

Attention deficit hyperactivity disorder (ADHD) is a prototypical neurodevelopmental disorder characterized by persistent symptoms of inattention, hyperactivity, and impulsivity. Although it typically manifests during childhood, these symptoms often persist into adolescence and adulthood. ADHD significantly impacts academic performance and daily functioning, primarily due to difficulties in sustaining attention, and is closely associated with various cognitive deficits^1,2^.

Theoretically, deficits in attention and impulsivity associated with ADHD have been attributed to impairments in executive functions^3^. Executive function refers to higher-order cognitive processes that regulate goal-directed behavior by integrating stimuli and controlling responses. These processes include stimulus prioritization, response inhibition, and cognitive shifting to achieve specific goals^4^. Barkley (1997) notably suggested that reduced prefrontal cortex activity in children with ADHD contributes to impairments in behavioral inhibition and attentional shifting, leading to difficulties in suppressing responses to irrelevant stimuli and producing abnormally short or prolonged reaction times^5–7^.

Among executive functions, attentional shifting has emerged as an important cognitive distinction between children with ADHD and typically developing (TD) peers^8–10^. Prior studies have consistently reported that children with ADHD show greater difficulties in inhibition and attentional shifting tasks compared to TD children. Notably, these differences in attentional shifting tend to persist with age^8^.

Traditional assessments of attentional shifting have primarily relied on set-shifting tasks such as the Wisconsin Card Sorting Test and the Trail Making Test-B^11^. These tasks typically require response changes based on rule shifts or selection among various stimuli, making them more complex than other cognitive tasks, and allowing researchers to estimate attentional shifting ability through measures of accuracy and reaction time (RT). Such complex conditions are utilized because simpler tasks may fail to detect behavioral differences between the ADHD and TD groups^12^. However, because these tasks engage other high-level cognitive functions, such as working memory, reasoning, and problem-solving, they provide limited insight into attentional shifting at a more fundamental level.

Low-level attentional shifting functions as a foundational mechanism for set shifting, which refers to higher-level attentional shifting that enables individuals to switch between rules or task sets^13^. Attentional shifting has three basic stages: orienting to sensory events, detecting signals for focal processing, and maintaining an alert state^14–16^. These processes together support the ability to flexibly shift mental sets in response to environmental changes, ultimately involving activation of the prefrontal cortex. However, conventional set-shifting tasks are more suited for assessing integrated executive functioning under cognitively demanding conditions and are therefore less effective for isolating early-stage orienting mechanisms^11,17^. To better understand the behavioral mechanisms underlying complex attentional shifts, it is essential to examine attentional shifting at this more basic level.

The gaze-cuing task provides a simplified paradigm for studying low-level attentional shifting by presenting a cue followed by a target. This task has been widely used to assess attentional mechanisms in children with Autism Spectrum Disorder (ASD)^18,19^. Because individuals with ASD often show reduced responsiveness to social cues, tasks including both social and non-social conditions are commonly used, allowing researchers to identify specific difficulties related to social attention. This task has also been adapted for ADHD studies, as children with ADHD often demonstrate reduced responsiveness to social cues and frequently exhibit comorbid features with ASD^18,20–23^. Several adaptations manipulating stimulus onset asynchrony (SOA) have revealed distinct patterns of reflexive and voluntary attention in children with ADHD compared to typically developing children^24^. Most previous studies have focused on behavioral indices, such as key responses, to evaluate whether participants responded to social cues. However, relying solely on key-press responses limits a comprehensive understanding of the attentional shifting process.

The introduction of eye-tracking technology into gaze-cueing tasks has enabled more detailed assessments of attentional processes, including eye movements and fixation patterns^22,23^. For instance, several studies have analyzed fixation durations within cue areas to examine gaze responsiveness^22^. Nevertheless, these studies primarily focus on cue utilization prior to target detection, rather than capturing the attentional shifting that occurs during early-stage orienting behavior in attentional shifts. While some studies have used various tasks, such as memory tasks, to differentiate ADHD, studies specifically targeting attention movement within gaze-cueing tasks remain limited^23^.

Although most previous studies have focused on higher-level attentional shifting within complex tasks^8–10^, the present study aimed to reveal subtle delays in low-level attentional shifting in children with ADHD using a simplified gaze-cueing task with minimal complexity. This task elicits joint attention by requiring coordination of gaze between a social cue and an object. A subtle congruent–incongruent manipulation with a distractor at the cue location was included to assess distraction susceptibility under minimal task demand. Unlike previous tasks that included multiple distractors, the use of only one distractor in this design allows for clear observation of simple attentional shifts in empty visual space. Difficulties in shifting higher-level attention, a deficit of set shifting often associated with ADHD^25^, may extend to low-level attentional processes. This can manifest as delayed eye movements during target detection or even a lack of eye movement altogether, where participants respond using peripheral vision without watching the target directly. Therefore, this study aims to investigate low-level attentional shifting characteristics through gaze behavior in children with ADHD using a simplified gaze-cueing task with reduced distractors. By analyzing both gaze movements and behavioral response data during a straightforward goal-oriented task, we seek to determine whether children with ADHD exhibit slower orienting behavior compared to TD children.

The objective of this study is to identify characteristic differences in attentional shifting between children with ADHD and TD children using a low-difficulty gaze-cueing task. Specifically, we aim to determine whether there are group differences in low-level attention shifts under conditions with minimal distractors, assessed through behavioral responses and eye-tracking data.

To this end, the following hypotheses were formulated:

1. In a simple gaze-cueing task, eye movement data will more effectively differentiate children with ADHD than key-press-based behavioral data.
2. Children with ADHD will exhibit less efficient attention shifting behavior compared to TD children.

For Hypothesis 1, behavioral and eye movement measures were collected to examine differences according to ADHD traits and task conditions, including cue type (social or non-social), target congruency, and stimulus onset asynchrony (SOA), using a linear mixed model (LMM). Indicators were identified based on significant effects in any condition. Feature selection was then performed, and models using either behavioral or eye movement features were compared.

For Hypothesis 2, orienting indicators identified from the analysis of Hypothesis 1 were incorporated into a logistic regression model to evaluate model performance. Additionally, an LMM was used to determine whether these indicators were associated with ADHD trait itself or other demographic or comorbid symptoms.

## Materials and methods

### Participants

A total of 44 TD children and 28 early elementary school-aged children with ADHD, aged 6–9 years, were initially recruited between August 2021 and February 2022. Although it is generally recommended to collect more than 30 samples per group based on the central limit theorem^26^, several previous studies comparing eye movements between children with ADHD and typically developing (TD) children have used smaller sample sizes^27,28^. In line with these precedents and due to practical constraints, we collected fewer than 30 samples for the ADHD group, ensuring that the effect size remained sufficient. TD children were recruited through an online community and local flyers, whereas children with ADHD were recruited from Korean medical centers and university hospitals. Eligibility was determined using an online application form that included exclusion criteria to ensure participants met the inclusion requirements.

All participants except two in the ADHD group had recently been diagnosed by a psychiatrist and had been on ADHD medication for less than one year. During participant recruitment, children exhibiting overt hyperactivity were excluded not only to prevent easy identification of their ADHD status, which could compromise the study design, but also due to ethical concerns regarding their potential discomfort or difficulty in completing the task. Participants with visual acuity below 0.3 on the decimal scale were excluded, as reduced acuity could interfere with accurate perception of visual cues during the task, potentially affecting their eye-movement patterns. Additional exclusion criteria included diagnoses of other developmental disorders, psychiatric illnesses, congenital genetic conditions, neurological diseases, or a history of acquired brain injury.

Further exclusions were made during data processing. Five children diagnosed with both ASD and ADHD were excluded to eliminate potential confounds from comorbid developmental disorders. Additionally, participants with an intelligence quotient below 70, as assessed by the Wechsler Intelligence Scale for Children—Fourth Edition, were excluded to control for borderline intellectual disability. Previous studies suggest that scores more than 1 standard deviations from the population mean are likely to indicate tendency of clinically and functionally abnormalities^29^. In a previous study^30^, the average inattention scores for the ADHD and TD groups were 11.22 (SD = 5.17) and 6.89 (SD = 3.67), respectively. ADHD participants with inattention scores 1 SD below the group mean (≤ 5) were excluded. TD children were excluded if their inattention score exceeded 1 SD above the TD group mean (≥ 10), or if their total K-ARS score exceeded the typical age-normed range (≥ 18, corresponding to 1 SD above the normative average).

**Fig. 1:**
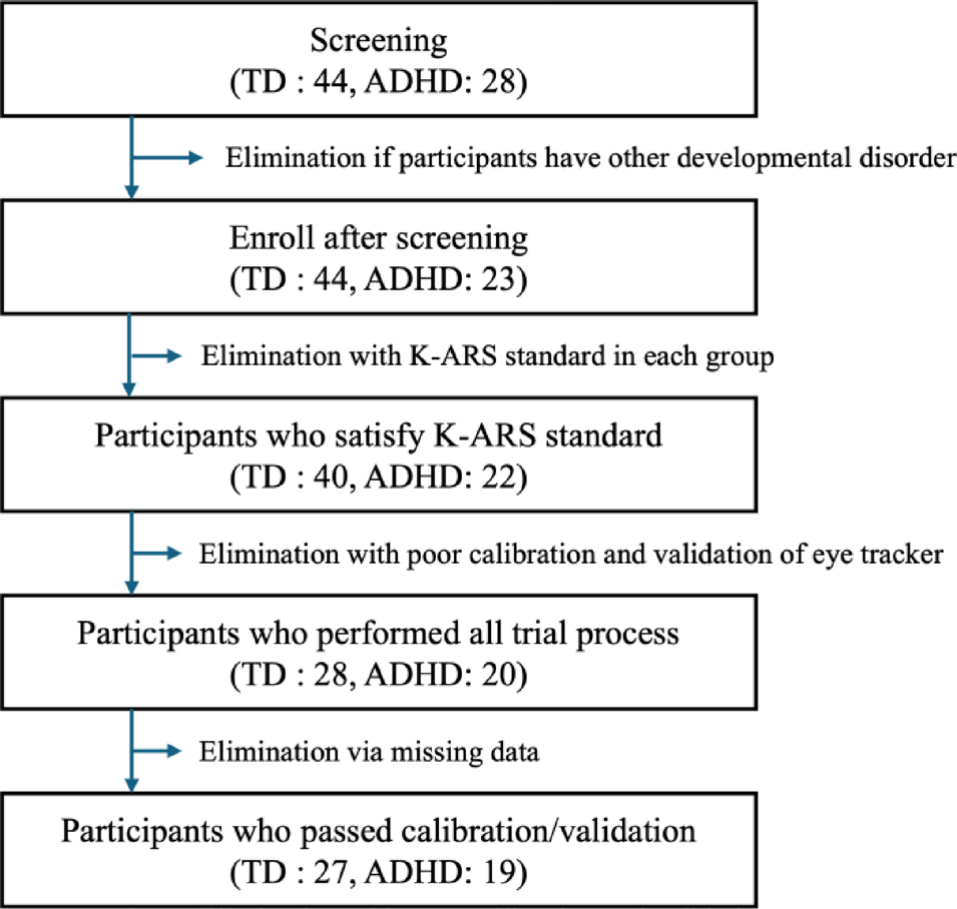
Flowchart of study participants and eligibility.

Participants were excluded if they failed the eye-tracker calibration (*n* = 9) or if their average visual deviation exceeded 2° during the validation trial (*n* = 5), even if initial calibration was successful. Although no sessions were terminated due to participant difficulty, data from one child in each group were lost due to equipment failure and were excluded. Figure 1 presents the overall flow chart.

The final sample consisted of 45 children: 19 with ADHD and 27 TD children. Among the children with ADHD, 12 had never taken medication, while the others had been on medication for periods ranging from 7 days to 2 years and 4 months. To control for the effects of medication, all participants were required to refrain from taking their medication on the day of the experiment. This study was approved by the Central Research Facilities Research Ethics Board of the Ulsan National Institute of Science and Technology (UNISTIRB-20–62-A). All research procedures were performed in accordance with the relevant guidelines and regulations, including the Declaration of Helsinki. Written informed consent was obtained from the parents or legal guardians of all participants prior to their participation in the study.

### Measurement

Additional exclusion criteria were implemented through self-reported surveys and clinical diagnoses to ensure eligibility in both clinical and nonclinical groups. The KARS was used as a primary screening tool. The scale includes two subscales: inattention and hyperactivity/impulsivity. As the study focused on recruiting children with ADHD who exhibited minimal hyperactivity, inattention scores were used as the primary screening criterion as described in *2.1. Participants*. KARS scores were also used as predictor variables to assess group differences.

The Childhood Autism Rating Scale (CARS) was used to screen for autism-related symptoms that might affect performance on the eye-tracking task (Cronbach’s alpha = 0.87). CARS is a widely used screening tool applicable regardless of age or cognitive ability. Participants with a total score of 37 or higher were considered to be at high risk for autism and were excluded from the study^31^. However, none of the participants exceeded this cutoff.

Kovac’s Children’s Depression Inventory (CDI) was used to assess baseline levels of depression^32^, and the State-Trait Anxiety Inventory for Children (STAI-C) was used to measure anxiety^33^. The CDI, adapted for children aged 8–13, comprises 27 multiple-choice items rated 0 to 2. It comprises 27 multiple-choice items, each rated on a scale from 0 to 2. A total score of 22 or above indicates mild depression, 26 or above indicates moderate depression, and 29 or above indicates severe depression. The Cronbach’s alpha for the CDI in previous studies was 0.898^34^. The STAI-C measures anxiety in children using 20 items each for state and trait anxiety. In this study, we used a validated Korean version adapted for elementary school students. Each item is rated on a scale from 1 (not at all) to 3 (often), with higher scores indicating greater anxiety levels (Cronbach’s alpha = 0.892)^34^.

Behavioral data were collected through keyboard inputs, measuring RT for each trial when the participant pressed a key corresponding to the target’s location and accuracy. Eye-tracking data were collected using a Tobii TX 300 eye tracker with a 300 Hz sampling rate. The monitor was 23”, with a screen resolution of 1920 × 1080, and data were collected using the Titta toolbox supported by the Tobii Pro SDK in MATLAB^35^. Eye movement data were smoothed using the Savitzky–Golay filter (sgolayfilt) with a polynomial order of 9 and a window size of 21. Eye movements with velocities ≥ 30°/s were classified as saccades, and those < 30°/s were classified as fixations. The distance between the participant’s eyes and the monitor was maintained at 65 cm using a chin rest. The cue, target, and distractor were presented at the four corners of the screen, each positioned 0.2 x-units and 0.2-y units from the center, with a diameter of 77 pixels.

### Material-gaze-cueing task

Before starting the task, the experimenter verbally explained the procedure to the participant. The task was introduced as a ‘red-dot finding game.’ The entire sequence—from the fixation point to the appearance of the face or arrow cue—was demonstrated. Children were instructed that a red dot would appear in one of the four corners of the screen. They were told to look at the center where the cue appeared, then shift their gaze to the red dot when it appeared, and press the button corresponding to its location. The instructions were repeated if the child did not fully understand them.

**Fig. 2:**
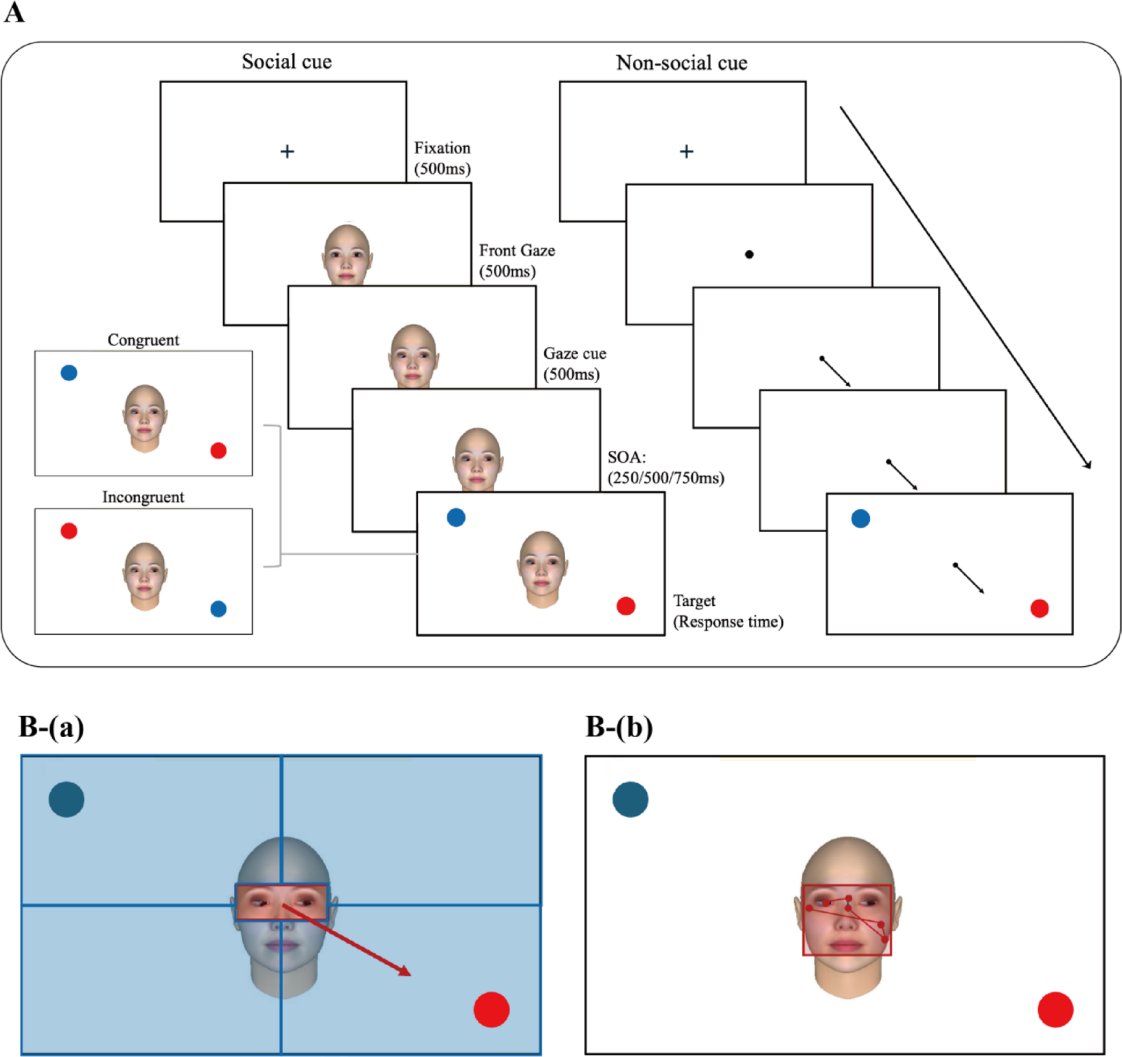
Overview of Experimental Task Involving Joint Attention and Peripheral Vision. A Sequence of gaze-cueing task. (**B**-(**a**)) Area of interest (AOI) to determine Response to joint attention (RJA) (**B**-(**b**)) peripheral vision occurrence.

To design a task related to joint attention (JA), we adapted Friesen and Kingstone’s gaze-cueing paradigm^36^. In this task, social stimuli (faces) and nonsocial stimuli (arrows) were used to cue the target either congruently or incongruently (Fig. 2). A cue appeared at the center of the screen and then shifted to point to one of the four corners for 500 ms. The target appeared following a randomized stimulus onset asynchrony (SOA) of 250, 500, or 750 ms. These SOA values were selected to capture different components of attentional orienting: shorter SOAs reflect rapid, reflexive orienting, whereas longer SOAs are associated with more intentional, voluntary shifts of attention^24^. Therefore, a range of SOAs was implemented to examine both reflexive and intentional aspects of attention in children. In the congruent condition, the cue directed attention toward the same quadrant as the target, whereas in the incongruent condition, a distractor appearing at the cue location pointed to the opposite quadrant. This manipulation enabled the assessment of distraction susceptibility under minimal task demand. The target was presented as a red dot, while a distractor (a blue dot) appeared simultaneously at one of the four corners to introduce interference. In the congruent condition, the distractor appeared in a location not aligned with the cue’s direction. In the incongruent condition, the distractor appeared in the direction of the cue to mislead the child’s attention. This design more effectively probed attentional shifting, as it required children to disengage from a cued distractor and reorient to the actual target, a process often impaired in children with ADHD. The target and distractor each subtended 2° of visual angle, with a consistent viewing distance of 65 cm from the screen.

To prevent directional bias, the direction of the cue and the locations of the target and distractor were randomized so that each combination appeared with equal probability. The distractor was randomly positioned in one of the three corners of the screen not occupied by the target. Within each block, congruent and incongruent trials were presented in random order, with equal frequency. Similarly, the three SOA conditions (250, 500, and 750 ms) were presented in equal proportions (1:1:1), ensuring the same number of trials for each condition. The entire task lasted approximately 15 min. There were four blocks, each consisting of 36 trials, and each block took about 3.6 min to complete. Social and nonsocial cues alternated between blocks, and the number of congruent and incongruent trials was evenly distributed within each block. The block sequence alternated between participants based on their order of arrival, such that one participant completed the social blocks first and the next completed the nonsocial blocks first. A break of approximately 1 min was provided after the completion of the first two blocks (between block 2 and 3). The congruency and SOA condition was appeared randomly in each block.

Before the start of blocks 1 and 3, a 6-point calibration task was performed to set up the eye tracker. Calibration was performed using the Tobii Pro Eye Tracker Manager software (https://developer.tobiipro.com/eyetrackermanager/etm-installation-information.html). Following calibration, a validation task was conducted to confirm calibration accuracy. A point was considered valid if its accuracy was within 2° of visual angle. Validation included three points; data were excluded if more than one point failed. Based on this criterion, 4 TD and 1 children with ADHD were excluded. The average validation accuracy among included participants was 1.84° for TD and 1.57° for ADHD children.

### Data analysis

This study extracted both behavioral and eye-tracking features. Behavioral features focused on accuracy and RT, which are commonly used metrics in previous studies^37^. RT was defined as the median duration between the onset of the target and the participant’s keyboard response, regardless of response accuracy. RT_correct, defined as the reaction time from correct trials, was used to verify intentional responses. Accuracy was defined as the proportion of trials in which the participant pressed the key corresponding to the correct target location (Multimedia Appendix). The number of saccades in this study was measured from the onset of the target to the participant’s response. All outcome measures—including saccades, reaction time, and fixation duration—were calculated within this target detection period to directly reflect attention-shifting behavior. Additionally, RT variability, an indicator of sustained attention, was computed as the SD of RT values^10^. The definitions and descriptions of eye-tracking features are provided in Table 1 in Multimedia Appendix.

Response to joint attention (RJA) was defined using Area Of Interest (AOI) centered on the cue (that is, the eye stimuli), and the surrounding screen was divided into quadrants, as illustrated in Fig. 2B-a. A gaze was classified as JA if it shifted from the central cue AOI to the quadrant indicated by the cue during the target detection period, without first entering another quadrant (Fig. 2B-a). Each trial was counted as a binary indicator, depending on whether a response to joint attention (RJA) occurred or not.

If the participant’s gaze remained fixed on the facial stimuli during the target detection period, without active gaze shifts toward the target location, this behavior was interpreted as target detection via “peripheral vision” (Fig. 2B-b). Peripheral-vision use was assessed in all trials regardless of button-press accuracy, as this measure aimed to capture an overall inattention-related eye-movement pattern, including cases where targets were detected without an overt gaze shift. RJA and the rate of peripheral vision behaviors were assessed only in blocks involving social stimuli.

To reduce the impact of outliers, continuous variables (except those capturing behavior occurrence) were computed using median values rather than mean values. For instance, the RT metric for social cues in the congruent condition at an SOA of 0.5 s was calculated as the median RT value for that specific condition. For binary variables (0 or 1), e.g., RJA presence or peripheral vision usage, mean values represented the proportion of trials exhibiting each behavior.

The gaze-cueing effect (GCE) was computed by subtracting the values for congruent stimuli from those for incongruent stimuli under each SOA condition during the target detection phase, in trials with social stimuli^37^. For example, GCE in RT at an SOA of 0.5 s was calculated by subtracting the RT under the congruent condition from that under the incongruent condition, both for social cues at that specific SOA.

To assess the appropriateness of the participant pool and identify group-level differences, sociodemographic and clinical characteristic data were analyzed using the Mann–Whitney U rank test, a nonparametric method used to compare the distributions of variables between ADHD and TD groups^38^.

For Hypothesis 1, indicators were calculated across each condition, including cue type (social or non-social), target congruency, and stimulus onset asynchrony (SOA), to examine which variables significantly differed between the ADHD and typically developing (TD) groups. Demographic variables including sex and age and psychological factors such as depression, anxiety, and autism traits (measured through self-report surveys) were controlled as covariates. A linear mixed model (LMM) was applied, with experimental conditions and their interactions specified as fixed effects and participant ID as a random effect. The analysis was conducted using the smf function from the *statsmodels* Python library. Before analysis, all continuous predictors were z-transformed to prevent coefficient estimates from being affected by differences in variable scales.

For indicators that showed a p-value below 0.15 in the fixed effects or psychological factors^39^, Mann–Whitney U rank test was employed to identify features with statistically significant differences between the groups to be used for classification. The indicators which is related to other psychological factors were also included because ADHD trait is highly comorbid to other psychiatric symptoms such as ASD, depression and anxiety so that the metric can be used for important discriminator for the classification model. To correct for multiple comparisons, the Benjamini—Hochberg procedure was applied using the *multipletest* function from the *Statsmodels* library in Python^40^. Only features that remained significant after corrections were retained for further analysis.

Next, stepwise logistic regression was conducted for feature selection using the StepReg library in R Studio^41^. This method employed bidirectional elimination, where variables were included based on their statistical significance. The entry and exit criteria for variable selection were based on Rao’s score test, with the significance threshold at 0.035. Both behavioral and eye-tracking features were subjected to this selection process, and the features identified as significant were used as inputs to train a combined predictive model.

Logistic regression modeling was then performed using selected features. To mitigate the risk of overfitting due to the small sample size, fivefold cross-validation was applied. Modeling was conducted in R Studio using the mlr3 package, and cross-validation was repeated 50 times to improve generalizability and reduce bias. During each iteration, label distribution was preserved across folds to maintain the balance between the ADHD and TD groups. For features found to be insignificant during modeling, feature importance was evaluated using the varImp function from the caret library in R Studio. Features with low importance scores were iteratively removed to determine whether this improved the performance of the model, particularly its area under the curve (AUC). This pruning process continued until no further AUC improvement was observed, resulting in the final feature set and model.

Model performance was evaluated based on the average accuracy, AUC, and F1 score (which incorporates both precision and recall) across the five validation folds. Consistency of the five cross-validation iterations was assessed using Cohen’s kappa coefficient, which measures the level of agreement between the actual labels and the predicted labels. Cohen’s kappa values were interpreted as follows: (0: Poor agreement; 0–0.2: Slight agreement; 0.21–0.40: Fair agreement; 0.41–0.60: Moderate agreement; 0.61–0.80: Substantial agreement; 0.81–1.00: Perfect agreement)^42^.

For Hypothesis 2, the average duration in each fixations during the target detection period, measured without predefined Areas of Interest (AOIs), was calculated to reflect natural gaze behavior during visual search. These values were derived from previously identified significant indicators and added to a logistic regression model to assess their contribution to model performance. To examine the association between this feature and ADHD traits, LMM was employed, using the same method of controlling for psychological variables as applied in Hypothesis 1.

## Results

### Sociodemographic and clinical characteristics of participants

The ADHD and TD groups were comparable in age, as both consisted of early elementary school–aged children. Although there was a difference in sex distribution, this disparity was expected due to the higher prevalence of ADHD diagnoses in males. The KARS, which measures ADHD symptom severity, revealed significant group differences in both the inattention and hyperactivity/impulsivity subscales. Additionally, scores on the CARS differed significantly, supporting prior research indicating that children with ADHD tend to exhibit more autistic traits than their TD peers^43^. No significant differences were found between the groups in state or trait anxiety. However, depression scores were significantly higher in the ADHD group (*p* =.003). Detailed sociodemographic information is summarized in Table 1.

**Table 1:** Sociodemographic and clinical characteristics of each group.

Measures (mean$\:\pm\:$SD)	ADHD (*n* = 19)	TD (*n* = 27)	*P* value
Age	7.7$\:\pm\:$1.0	7.9$\:\pm\:$0.9	0.709
Sex (male n, %)	16 (84.2)	12 (44.4)	-
KARS total score	22.4$\:\pm\:$11.2	$\:5.6\pm\:$4.4	< 0.001
Inattention score	13.7$\:\pm\:$6.0	3.5$\:\pm\:$2.8	< 0.001
Hyperactivity/impulsivity score	8.7$\:\pm\:$6.0	2.1$\:\pm\:$2.1	< 0.001
CARS	18.7$\:\pm\:$5.3	15.1$\:\pm\:$0.4	< 0.001
CDI	13.0$\:\pm\:$6.5	7.7$\:\pm\:$3.7	0.003
STAI-C			
Trait anxiety	30.3$\:\pm\:$5.8	28.3$\:\pm\:$5.2	0.259
State anxiety	33.3$\:\pm\:$6.9	30.6$\:\pm\:4.9$	0.176

### Hypothesis 1: during a simple gaze-cueing task, eye movement data can distinguish children with ADHD more effectively than behavioral data

LMM was conducted using both behavioral indicators (accuracy, reaction time [RT], and RT for correct responses [RT_correct]) and eye movement indicators (number of saccades, saccade length, saccade velocity, fixation duration on the cue during SOA, standard deviation of fixation locations, null data rate, and the rate of peripheral vision). Fixed effects included group (ADHD vs. TD), congruency, SOA, and cue type.

Among the behavioral indicators, both accuracy and RT showed significant group differences, with a significant interaction between group and congruency (*p* <.05). However, RT_correct did not show any significant fixed effects or associations with psychological scales; only age showed a significant effect (*p* <.001).

Regarding eye movement indicators, fixation duration on the cue, saccade velocity, and the rate of peripheral vision usage showed significant main effects of group or significant interactions with other fixed effects (*p* <.05). Congruency showed a marginal effect on joint attention (*p* =.099), but was significantly associated with saccade length and the standard deviation of fixation locations (*p* <.001). The null data rate did not show any significant fixed effects, although depressive symptoms measured by the CDI were significantly associated with this rate (*p* =.043).

LMM results, including coefficients and p-values (for *p* <.1), are presented in Appendix 2.

The Mann–Whitney U test was used to compare group differences across indicators that LMM show its significance in fixed effect or psychological factors. Accordingly, RT_correct, which showed no significant effects either in fixed effects or psychological factors, was excluded from the feature selection process. As summarized in Table 2, trials using social cues showed significant group differences in the number of saccades, saccade velocity, and the SD of fixation locations. Approximately half of the significantly different features were related to RJA.

The frequency of RJA was higher in the TD group compared to the ADHD group. Conversely, the difference in the number of saccades between incongruent and congruent conditions was larger in the ADHD group, with this contrast reaching high significance (*p* <.001).

In trials utilizing nonsocial cues, significant indicators included the number of saccades, saccade velocity, SD of fixation locations, saccade length, and the ratio of null data (Table 2).

**Table 2:** Distribution of eye-tracking features between ADHD and TD groups, based on variables identified as significant in the Mann–Whitney U test.

Stimuli	Data sort	Feature (SOA/congruency)	ADHD	TD	Cohen’s D	*p*-value
Social	Eye movement	Number of saccades (0.25 s/cong)	6.89 ± 6.03	10.3 ± 5.5	−0.594	0.043
		Number of saccades (0.5 s/cong)	5.89 ± 4.33	10.46 ± 6.09	−0.84	0.037
		Number of saccades (0.75 s/cong)	6.66 ± 5.57	9.69 ± 5.49	−0.548	0.043
		Saccade velocity (0.5 s/incong)	89.56 ± 22.18	100.09 ± 24.45	−0.447	0.043
		Saccade velocity (0.75 s/incong)	89.78 ± 21.21	100.08 ± 25.84	−0.428	0.045
		Standard deviation (SD) of fixation locations (0.25 s/cong)	4.91 ± 3.16	6.29 ± 3.1	−0.443	0.045
		Rate of response to joint attention (RJA) (0.25 s/cong)	0.24 ± 0.14	0.34 ± 0.1	−0.804	0.043
		Rate of RJA (0.25 s/incong)	0.2 ± 0.13	0.3 ± 0.11	−0.834	0.037
		Gaze cuing effect (GCE) in the saccade number (0.5 s/incong–cong)	3.13 ± 2.99	−0.57 ± 3.02	1.233	0.009
		Rate of peripheral vision (0.25 s/incong)	0.64 ± 0.16	0.56 ± 0.11	0.627	0.046
	Behavior	GCE in reaction time (RT) (0.5 s/incong–cong)	0.12 ± 0.15	0.03 ± 0.07	0.855	0.045
Non- social	Eye movement	Saccade length (0.75 s/cong)	11.3 ± 6.02	16.59 ± 8.32	−0.708	0.041
		Saccade length (0.75 s/incong)	20.12 ± 13.15	27.02 ± 10.67	−0.588	0.043
		Number of saccades (0.25 s/cong)	5.71 ± 4.53	9.22 ± 5.5	−0.685	0.037
		Number of saccades (0.25 s/incong)	6.61 ± 4.72	10.81 ± 6.28	−0.739	0.037
		Saccade velocity (0.5 s/incong)	96.97 ± 23.09	105.08 ± 21.53	−0.366	0.043
		Saccade velocity (0.75 s/cong)	83.94 ± 18.39	94.76 ± 19.11	−0.575	0.043
		Saccade velocity (0.75 s/incong)	93.67 ± 20.15	104.71 ± 23.69	−0.495	0.037
		SD of fixation locations (0.25 s/incong)	7.13 ± 3.46	9.08 ± 3.11	−0.596	0.043
		SD of fixation locations (0.5 s/incong)	7.56 ± 3.43	9.43 ± 3.3	−0.557	0.043
		SD of fixation locations (0.75 s/incong)	6.39 ± 3.76	9.3 ± 3.51	−0.805	0.037
		Rate of null data (0.5 s/cong)	0.07 ± 0.13	0.0 ± 0.02	0.841	0.041
		Rate of null data (0.75 s/cong)	0.08 ± 0.1	0.01 ± 0.06	0.808	0.043
		Rate of null data (0.75 s/incong)	0.05 ± 0.09	0.0 ± 0.0	0.801	0.043
	Behavior	Accuracy (0.25 s/incong)	0.76 ± 0.35	0.98 ± 0.03	−0.981	0.037
Total	Eye movement	Rate of null data	0.11 ± 0.1	0.05 ± 0.04	0.957	0.041
		SD of null rate	0.15 ± 0.09	0.1 ± 0.06	0.727	0.043
	Behavior	SD of RT	0.55 ± 0.52	0.26 ± 0.17	0.818	0.043

Overall, the TD group exhibited more frequent and faster eye movements during the target detection period, along with greater variability in gaze behavior.

Three behavioral features were significantly different between the groups. Accuracy under the nonsocial cue condition at an SOA of 0.25 s during incongruent trials differed significantly (*p* =.008), whereas accuracy under other conditions did not. The SD of RT differed significantly across all conditions (*p* =.033), and the GCE in RT at an SOA of 0.5 s also showed a significant group difference (*p* =.044).

Stepwise regression analysis identified accuracy and GCE in RT as significant predictors in the behavioral model. For the eye movement model, key features included the number of saccades, GCE in number of saccades, RJA rate, and the proportion of null data. Notably, approximately half of the selected features in both models were related to RJA.

Irrespective of the data source, the model included the SD of the RT, number of saccades, GCE in the number of saccades, and SD of fixation locations when considering the entire dataset.

Model performance metrics derived from logistic regression using these features are presented in Table 3.

**Table 3:** Features used for logistic regression and model performance by each dataset.

Dataset	Features used for the model	Kappa	Acc	F1 score (precision/recall)	AUC
Behavior	Accuracy (0.25s/incong)^*^ Gaze cuing effect (GCE) in RT (0.5)^*^	0.435	0.741	0.788 (0.746/0.871)	0.828
Eye movement	Number of saccade (0.25s/incong)^*^ GCE in number of saccade (0.5s)^*^ Rate of response to joint attention (0.25s/cong) Rate of null data^*^	0.644	0.837	0.853 (0.863/0.872)	0.929
Total	SD of reaction time (RT)^*^ Number of saccade (0.25s/cong)^*^ GCE in number of saccade (0.5s)^*^ SD of fixation locations (0.25s/incong)^·^	0.716	0.870	0.882 (0.883/0.901)	0.911

^*^ Indicates significant variables in the logistic regression (*p* <.05).

The eye movement model outperformed the behavioral model in both accuracy (0.837 vs. 0.741) and AUC (0.929 vs. 0.828). While the accuracy and AUC of the eye movement model were comparable to those of the combined model, the kappa statistic was higher for the integrated model that incorporated both behavioral and eye-tracking data (kappa = 0.716). The behavior-only model demonstrated a moderate level of agreement (k = 0.435), whereas both the eye movement-only and combined models achieved significant agreement according to established kappa interpretation guidelines^42^.

### Hypothesis 2 : under minimal distractor interference, children with ADHD show less efficient goal-oriented attentional shifting compared to TD children

**Fig. 3:**
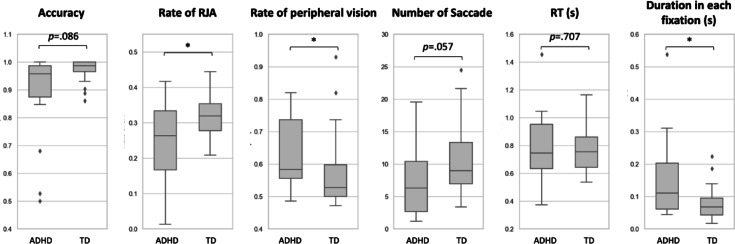
Feature exploration during target detection with social cues, irrespective of SOA and congruency; RJA : Response to joint attention; RT : Reaction time.

Although RT did not differ significantly between the groups (*p* =.707), a trend emerged suggesting that the TD group produced a higher number of saccades (*p* =.057, d= −0.516). This trend prompted an examination of duration in each fixation during target detection, calculated by dividing total fixation duration by the number of fixation during target detection. Results showed that children with ADHD had significantly longer duration in each fixation during target detection compared to TD children (*p* =.017, d = 0.799), indicating slower reorientation of gaze during the target-search process. However, accuracy was lower in the ADHD group compared to the TD group (*p* =.086). Figure 3 illustrates the distributions of these features.

Following the inclusion of eye movement metrics in the subset selection process, model performance remained consistent regardless of whether features were selected from eye-tracking data alone or in combination with behavioral data. The key features identified included:

(1) GCE in number of saccades at SOA = 0.5 s, (2) rate of null data under nonsocial cues (SOA = 0.5 s, incongruent), and (3) duration in each fixation during target detection in social cue trials. Notably, the “duration in each fixation during target detection” was a novel metric identified specifically under social cue conditions during the target detection period. Incorporating this metric improved the performance of the logistic regression model. The updated model achieved an AUC of 0.940 and an accuracy of 0.860, demonstrating a significant improvement over the previous version (Table 4).

**Table 4:** Comparison of the model with and without duration in each fixation.

		Duration in each fixation -			Duration in each fixation +	
		β	Z¶ (*p*-value)	β		Z¶ (*p*-value)
Features	SD of reaction time (RT)	14.771	2.467 (0.014)			
	Number of saccade (0.25 s/cong)	− 0.842	−2.348 (0.019)			
	SD of fixation locations (0.25 s/incong)	− 0.666	−1.676 (0.094)			
	Gaze cuing effect in number of saccade (0.5 s)	1.901	2.177 (0.030)	1.306		2.485 (0.013)
	null_rate in nonsocial (0.5 s/cong)			114.266		2.449 (0.014)
	Duration in each fixation during target detection			43.157		2.646 (0.008)
Kappa		0.716			0.696	
Acc		0.870			0.860	
F1 score(precision/recall)		0.882 (0.883/0.901)			0.876 (0.879/0.891)	
AUC		0.911			0.940	

.

The correlations between selected model features and the ADHD symptom severity, as measured by the KARS, were evaluated (Table 2 in Multimedia Appendix). Duration in each fixation during target detection exhibited the strongest correlation with inattention (*r* =.46, *p* <.01) and hyperactivity scores (*r* =.36, *p* <.01). Features such as GCE in number of saccades at an SOA of 0.5 s and the rate of null data in nonsocial cues at an SOA of 0.5 s under incongruent conditions showed a significant correlation with the inattention KARS scores (*p* <.01). Conversely, among other features, only the SD of RT showed a significant correlation with the hyperactivity KARS scores (*r* =.32, *p* <.01), excluding the duration at each fixation point. Other features included in the model did not show statistically significant correlations with KARS scores (Table 2 in Multimedia Appendix).

**Fig. 4:**
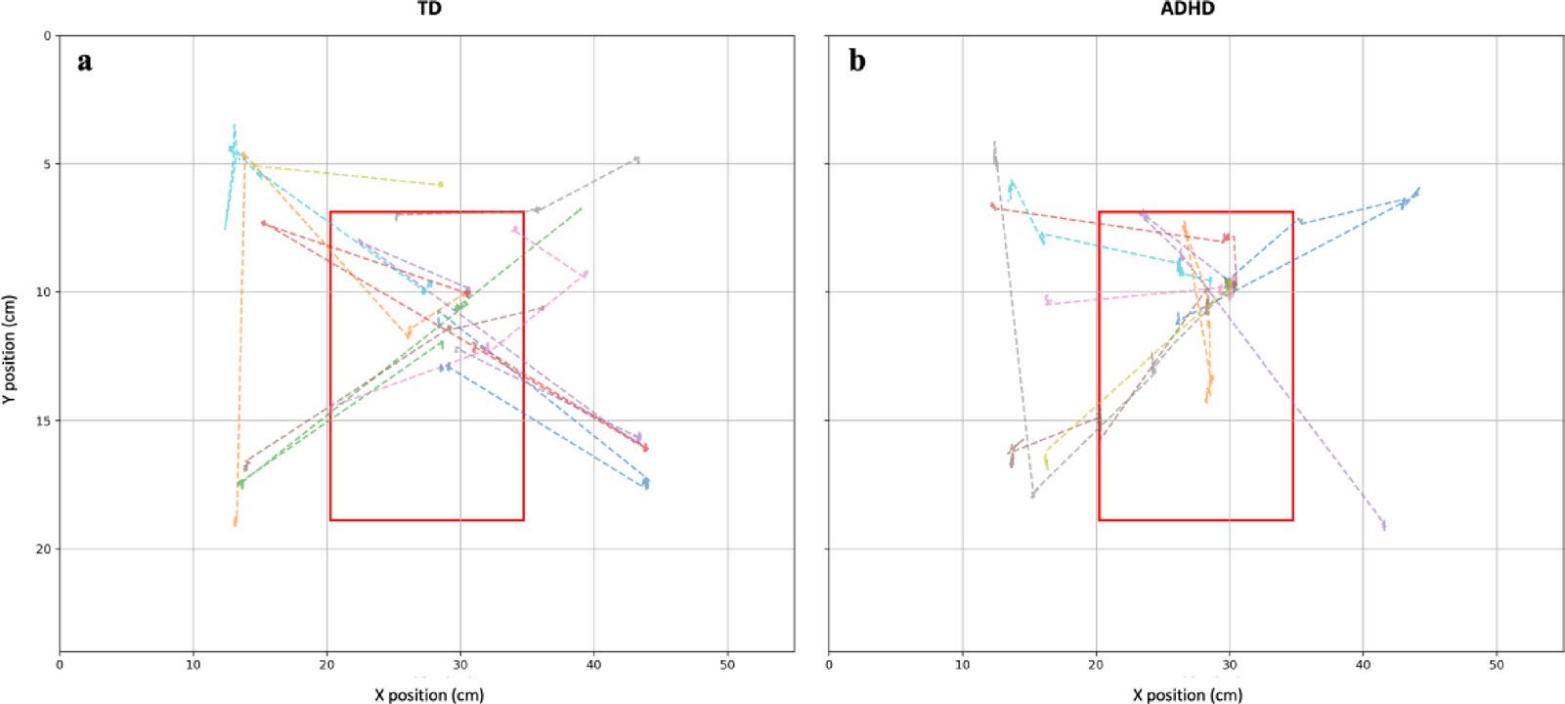
Eye movement pathways from cue to target detection across 10 trials. (**a**) Eye movement trajectory of one participant from the TD group during 10 trials of the gaze-cuing task; (**b**) Eye movement trajectory of one participant from the ADHD group during the same task.

In trials involving social cues, the rate of RJA was significantly higher in the TD group than in the ADHD group (*p* =.042, d = −0.827), regardless of SOA or cue congruency. Furthermore, despite comparable RTs, the ADHD group demonstrated significantly longer durations per each fixation in target detection than the TD children (*p* =.017, d = 0.799). Figure 4 shows representative eye movement patterns for the two groups.

These findings suggest that children with ADHD were more likely to remain at their initial fixation location during target detection, rather than promptly redirecting their gaze toward the target.

Additionally, the ADHD group exhibited a significantly higher rate of peripheral vision use during the target detection period compared to the TD group, as illustrated in Fig. 3 (*p* =.046, d = 0.481).

To investigate the underlying factors influencing fixation duration during target detection and the rate of peripheral vision, a linear mixed model (LMM) was conducted with group and task condition as fixed effects, and psychological traits and demographic variables as covariates. Interaction terms between fixed effects were not significant. Group differences were significant for both fixation duration (*p* =.032) and the rate of peripheral vision (*p* =.025) during target detection. For the rate peripheral vision, trait anxiety as measured by STAIC was also a significant predictor (*p* =.001). Other experimental conditions did not show significant effects. When fixation duration was analyzed, group was the only significant factor; no other covariates yielded significant coefficients. Detailed results are provided in Appendix X.

To further examine the effect of individual traits, the KARS score was included in place of the group variable. The results, presented in Table 5, show that KARS significantly predicted fixation duration (β = 0.039, *p* =.005), while other variables were not significant. For the rate of peripheral vision, both KARS (*p* =.014) and CARS (*p* =.022), which reflects ASD traits, were significant predictors. Trait anxiety remained a significant factor (*p* =.001) for the rate of peripheral vision. Both models showed good explanatory power. The ratio of peripheral vision model had a marginal R² of 0.224 and a conditional R² of 0.620, and the duration in each fixation during target detection model showed similar strength (R²m = 0.241, R²c = 0.714).

**Table 5:** Linear mixed-effects model results for duration in each fixation during target detection and rate of peripheral vision.

Predictor	Duration in each fixation¶ (*R*^2^m = 0.241, *R*^2^c = 0.714)		Rate of peripheral vision¶ (*R*^2^m= 0.224, *R*^2^c = 0.620)	
	β	z (*p*)	β	z (*p*)
KARS	0.039	2.785 (0.005)	0.033	2.459 (0.014)
CARS	−0.182	−1.092 (0.275)	−0.362	−2.296 (0.022)
CDI	0.087	0.518 (0.604)	0.299	1.873 (0.061)
STAIC status	0.167	1.263 (0.206)	0.092	0.733 (0.463)
STAIC trait	−0.136	−1.028 (0.304)	−0.418	−3.34 (0.001)
Age	−0.112	−0.883 (0.377)	0.22	1.826 (0.068)
Sex (female)	−0.143	−0.543 (0.587)	0.198	0.796 (0.426)
Congruency	−0.145	−1.207 (0.228)	0.155	1.109 (0.267)
SOI 0.5	−0.071	−0.592 (0.554)	0.282	2.017 (0.044)
SOI 0.75	0.168	1.396 (0.163)	0.099	0.706 (0.48)

## Discussion

This study utilized a gaze-cuing task to collect both behavioral and eye movement data from children, with the aim of identifying indicators of ADHD and developing an early-stage classification model. Our findings supported all three hypotheses and provided new insights into low level of attentional and executive functioning deficits in children with ADHD, even in low-demand tasks.

First, consistent with our initial hypothesis, this study highlights the diagnostic utility of eye movement data in detecting ADHD, particularly in low-demand tasks where behavioral differences are subtle, although the current findings indicate a more nuanced interpretation. Unlike previous studies that reported significant differences in accuracy among children with ADHD^44^, we observed significant behavioral differences in accuracy only under the social cue condition with a SOA of 0.25 s and incongruent cues. Conversely, the model developed in this study achieved relatively high performance using only eye movement indicators (AUC = 0.929, accuracy = 0.837). These results suggest that the gaze-cueing task employed in this study may not have been sufficiently demanding to differentiate the groups based on behavioral indicators alone^43^.

Second, our findings support the hypothesis that children with ADHD exhibit less efficient goal-oriented attentional shifting, even in environments with minimal distractor interference. This was demonstrated by prolonged duration in each fixation during target detection and reduced saccadic frequencies during the target detection behavior. These eye movement patterns suggest delayed attentional disengagement from irrelevant areas and reduced engagement in active visual search—both of which are essential for effective attentional shifting. These findings align with prior research linking reduced saccadic frequency to impaired attentional control and suggesting that limited exploratory eye movements reflect executive dysfunction in ADHD^45^.

Moreover, duration in each fixation during target detection emerged as the most predictive feature in our classification model, showing strong correlations with KARS inattention (*r* =.46, *p* <.01) and hyperactivity scores (*r* =.36, *p* <.01). Additionally, this feature was uniquely associated with ADHD traits, regardless of comorbid psychological factors such as autism, depression, or anxiety, while other indicators were significant only in the LMM analysis. Prior meta-analyses have indicated that small to moderate deficits in attentional shifting are associated with inattention and hyperactivity^46^. Our findings further suggest that prolonged fixations reflect more than motor slowing—they may indicate a reduced capacity for flexible attentional engagement.

Finally, although peripheral vision use was significantly elevated in the ADHD group, it was also associated with traits related to autism and anxiety. This pattern may reflect heightened sensitivity to environmental stimuli, characterized by attentional or sensory hyper-responsiveness, which is commonly observed in individuals with ADHD and frequently overlaps with comorbid characteristics such as those seen in autism spectrum disorder and anxiety^47,48^.

While previous work has shown that ADHD may not impair set-shifting speed when high-level executive functions remain intact^25^, our findings suggest deficits at more automatic, early stages of orienting. This supports a hierarchical model of attentional control, where ADHD symptoms manifest at multiple cognitive levels. The subtle nature of these low-level impairments highlights the importance of examining nontraditional indicators, such as prolonged fixations, to capture executive dysfunction beyond what standard neuropsychological tasks can detect.

Third, our data support the hypothesis that children with ADHD rely more on peripheral vision than on active, intentional target detection. Compared with TD children, the ADHD group exhibited both a lower rate of RJA and a higher rate of peripheral vision use. JA serves as a regulatory mechanism that facilitates shared attention and social coordination^22,49^. A reduced rate of RJA in the ADHD group suggests impaired use of external cues for attentional guidance. This deficit may indicate overlapping impairments in social cue responsiveness, consistent with the high comorbidity between ADHD and ASD^50^. Given that reduced RJA is a prominent early marker for ASD, our finding of diminished RJA in children with ADHD supports the notion of shared difficulties in processing socially relevant cues^51^.

Although a previous study reported increased face fixation time in children with ADHD across the task duration^22^, this study adopts a more focused approach by examining eye movements immediately following gaze cue onset. This allowed us to assess the visual strategies employed specifically during goal-directed target detection. Our findings reveal that children with ADHD are more likely to rely on peripheral visual input and to engage in less structured visual scanning.

Notably, the ADHD group demonstrated a greater difference in saccade frequency between congruent and incongruent trials than the TD group. This pattern may reflect heightened sensitivity to conflicting cues, indicating reduced cognitive control in situations that demand attentional flexibility^25^.

Across all three hypotheses, eye movement patterns in the ADHD group consistently indicated delayed and inefficient attentional shifting, as well as difficulty using cues to guide attention. These impairments—evident even in a distraction-free, low-demand task—highlight fundamental executive dysfunctions in ADHD patients and suggest that deficits in attentional shifting impairments may serve as robust behavioral markers for early identification.

This study presents several notable strengths. First, it employed a simple gaze-cueing task devoid of complex distractors to investigate low-level attentional shifting in children with ADHD. Although such paradigms are commonly used in developmental research, they have rarely been applied to detect subtle attention deficits in ADHD populations. Despite minimal task demands, significant group differences emerged in eye-tracking measures.

Second, the study identified prolonged fixation duration during target detection as a novel and highly predictive marker of ADHD. This feature was linked to reduced attentional shifting efficiency and showed strong correlations with clinical indices of inattention and hyperactivity. Its inclusion in the classification model significantly improved predictive accuracy, underscoring its potential as a core feature for early ADHD detection.

Furthermore, by analyzing eye movement patterns, this study provided insight into the behavioral mechanisms underlying attentional shifts in ADHD. These findings revealed physiological indicators that may facilitate early identification, and could be integrated with other bio-signals—such as electroencephalography—to enhance diagnostic precision in future multimodal assessments.

Lastly, despite the limited sample size, the study achieved high classification accuracy and AUC values using a minimal set of eye movement features. The use of cross-validation techniques helped reduce overfitting, suggesting these indicators may scale well in larger diagnostic frameworks.

Unlike previous gaze-cueing studies that primarily analyzed cue utilization before target appearance, our study isolates attentional shifting during the target-detection window, revealing micro-level reorientation delays that were not detectable in prior designs.

Nevertheless, some limitations should be acknowledged. Although this study highlights the potential value of eye-movement data in low-demand tasks where behavioral differences are subtle, it remains challenging to draw a definitive conclusion that eye-movement features are more discriminative than behavioral measures. This limitation stems from the fact that behavioral indicators were available in a more restricted form, which may have constrained their discriminative capacity. a Furthermore, although prolonged dwell at non-target locations was interpreted as slower reorientation of gaze, it is also possible that some instances reflected mis-cueing rather than reduced shifting efficiency. Disentangling these mechanisms will require additional trial-level analyses, and this ambiguity has been noted as a limitation of the present study.

Another limitation is that the social (eye-gaze) and non-social (arrow) cues differed in their intrinsic visual form, making it difficult to match cue size perfectly across conditions. Although gaze cues generally elicit automatic orienting even at smaller scales, this unavoidable size discrepancy may still have influenced cue saliency.

Additionally, the small sample size may restrict the generalizability of the findings. This study also has a limitation in that it did not control for the duration of prior medication use. To minimize medication effects, we included 12 children who were newly diagnosed and had no prior experience with medication. However, other participants may still have been affected by previous medication use, despite refraining from taking it on the day of the experiment. To overcome those small sample size, I included two more participants who has been taken medicine for more than 1 year, but they Future studies with larger and more diverse samples are needed to validate and refine these findings.

Another limitation of this study is that cognitive evaluations were not conducted for the TD group. TD children were screened to rule out developmental or psychiatric disorders, which makes the likelihood of an IQ below 80 very low. Additionally, conducting a full standardized cognitive assessment would have required substantial additional testing time and imposed participant burden without clear clinical justification. Nonetheless, it remains possible that some children may have had lower cognitive functioning, and ideally, formal IQ assessments should have been included.

Also, there was the difference in calibration quality between groups. The TD group exhibited a higher rate of calibration failure because these participants were tested earlier in the study, before the experimenter had fully gained proficiency with the calibration procedure. Although this resulted in a higher dropout rate in the TD group, the final sample sizes remained sufficient for the planned analyses and were unlikely to have substantially affected the overall findings.

This study demonstrates that even a simple gaze-cueing task, when analyzed using a logistic regression model incorporating behavioral and eye movement data, can objectively detect atypical attentional shifting in children with ADHD. Unlike previous gaze-cueing studies that primarily focused on cue utilization before target onset, our approach isolates attentional shifting during the target-detection window, revealing micro-level reorientation delays that were not detectable in prior designs. Notably, eye-tracking indicators—particularly prolonged fixation durations during target detection and the use of peripheral vision—were more sensitive than traditional behavioral measures in distinguishing children with ADHD, emerging as strong markers closely associated with core symptoms. In particular, fixation duration during target detection demonstrated a unique association with ADHD traits, independent of comorbid symptoms such as autism, anxiety, or depression.

These findings underscore the potential of combining simplified gaze-cueing tasks with eye-tracking technology to reveal subtle executive dysfunctions and enable early, efficient ADHD screening. The strong classification performance further supports the utility of these markers. Future research should expand sample sizes and explore these markers across diverse populations and settings to enhance generalizability and refine diagnostic tools.

## Supplementary Information

Below is the link to the electronic supplementary material.

Supplementary Material 1

Supplementary Material 2

## Data Availability

The datasets generated and/or analyzed during this study are not publicly available due to participant confidentiality agreements, but are available from the corresponding author on reasonable request.
