# Supplementary material for: Exploring early-stage orienting behavior using an eye tracker for attention deficit hyperactivity disorder classification
Source: Sci Rep. 2026 Feb 26;16:8671. doi: 10.1038/s41598-026-41419-0 (PMC12979651; doi:10.1038/s41598-026-41419-0)
Supplement: Supplementary file 1 — Supplementary Material 1 [file 41598_2026_41419_MOESM1_ESM.pdf]

Table 1. Definition of features extracted from behavior and eye tracking data.

Feature	Description
**Behavioral feature**	
Accuracy	Number of button presses on the same position with the target
Reaction time (RT)	Duration from target appearance to pushing the button
SD of RT	Standard deviation of the reaction time across trials
**Eye movement features**	
Fixation duration	Fixation on cue stimuli in the area of interest before target appearance
SD of fixation locations	Standard deviation of fixation locations during the target detection period
Saccade velocity	Saccade velocity before target appearance
Number of saccades	Total number of saccades during the target detection period
Saccade velocity	Saccade velocity during the target detection period
Saccade length	Total length of saccade movement during the target detection period
Rate of null data	The rate of null samples across all eye tracker data
SD of null rate	Standard deviation of the rate of null data
Rate of joint attention (JA)	Number of joint attention occurrences
GCE in saccade number	GCE calculated by the difference between congruent and incongruent conditions of the saccade number during the target detection period
GCE in RT	GCE calculated by the difference between congruent and incongruent conditions of the RT during the target detection period
Rate of peripheral vision	Number of peripheral observations of target appearance

Table 2 Correlations between the KARS score and each feature selected for logistic regression.

Category	Indicators	1	2	3	4	5	6	7	8	9	10		
KARS score	1. Inattention												
	2. Hyperactivity	0.84***											
Social	3. Rate of JA (0.25-s SOA/cong)	-0.3**	-0.21										
	4. GCE in the number of saccades (0.5-s SOA)	0.35**	0.35**	-0.2									
	5. JA score in the RT (0.5-s SOA)	-0.02	-0.02	-0.25	0.61***								
	6. Number of saccades (0.25-s SOA/cong)	-0.23	-0.11	0.14	-0.0	-0.04							
	7. Duration of each fixation point	0.46**	0.36**	-0.22	-0.01	0.09	-0.66***						
Nonsocial	8. Number of saccades (0.25-s SOA/incong)	-0.39**	-0.35**	0.24	-0.12	0.09	0.68***	-0.49***					
	9. SD of fixation locations (0.25-s SOA/incong)	-0.23	-0.1	0.09	0.04	-0.09	0.37**	-0.45**	0.33**				
	10. null_rate (0.5-s SOA/incong)	0.36**	0.24	-0.11	0.17	0.28	-0.14	0.54***	-0.05	-0.17			
	11. Accuracy (0.25-s SOA/incong)	-0.29	-0.17	0.08	-0.27	-0.35**	0.13	-0.17	0.06	0.47**	-0.03		
Both	12. Rate of nulls	0.33**	0.24	-0.07	0.18	0.26	0.04	0.33**	0.01	-0.26	0.77***	-0.17	
	13. Variance of RT	0.27	0.32**	-0.09	0.28	0.39**	0.36**	0.0	0.26	-0.1	0.36**	-0.5***	0.63***

JA: Joint attention; SOA: stimulus onset asynchrony; RT: reaction time; SD: standard deviation; cong/incong: congruent cue/incongruent cues.

** indicates correlation is significant with p <.01

*** indicates correlation is significant with p <.001
