# Supplementary material for: Exploring early-stage orienting behavior using an eye tracker for attention deficit hyperactivity disorder classification
Source: Sci Rep. 2026 Feb 26;16:8671. doi: 10.1038/s41598-026-41419-0 (PMC12979651; doi:10.1038/s41598-026-41419-0)
Supplement: Supplementary file 2 — Supplementary Material 2 [file 41598_2026_41419_MOESM2_ESM.docx]

Table 2. Linear mixed-effects model results across all experimental conditions (significant coefficients only).

| **DV** | **variable** | **coefficient** | **z** | **p** |
| --- | --- | --- | --- | --- |
| accuracy | Group X Congruency | -0.882 | -2.923 | 0.003 |
|  | CDI | -0.572 | -6.464 | 0 |
|  | STAIC_trait | 0.185 | 2.711 | 0.007 |
|  | STAIC_status | 0.168 | 2.454 | 0.014 |
| RT | Congruency | 0.183 | 1.555 | 0.12 |
|  | Group X Congruency | 0.363 | 1.974 | 0.048 |
|  | Group X Cue type | -0.385 | -2.094 | 0.036 |
|  | CDI | -0.267 | -1.595 | 0.111 |
|  | age | -0.467 | -3.762 | 0 |
| RT_accurate | age | -0.137 | -3.92 | 0 |
| Number of saccades | Congruency | 0.201 | 1.45 | 0.147 |
|  | SOA(0.5) X Congruency | -0.301 | -1.538 | 0.124 |
|  | Group X SOA(0.5) X Congruency | 0.471 | 1.547 | 0.122 |
|  | STAIC_status | -0.199 | -1.586 | 0.113 |
| Saccade length | Congruency | 0.623 | 4.703 | 0 |
|  | Cue type | -0.327 | -2.47 | 0.014 |
|  | Group X SOA(0.75) X Cue type | -0.481 | -1.649 | 0.099 |
|  | CARS | 0.274 | 2.047 | 0.041 |
|  | CDI | -0.333 | -2.19 | 0.029 |
|  | STAIC_trait | 0.259 | 2.216 | 0.027 |
|  | age | -0.336 | -2.982 | 0.003 |
| Saccade velocity | Congruency | 0.223 | 1.621 | 0.105 |
|  | Group X SOA(0.5) | 0.316 | 1.482 | 0.138 |
|  | Group X SOA(0.75) | 0.386 | 1.809 | 0.07 |
|  | SOA(0.75) X Cue type | 0.365 | 1.881 | 0.06 |
|  | Group X SOA(0.75) X Congruency | -0.471 | -1.559 | 0.119 |
|  | Group X SOA(0.75) X Cue type | -0.777 | -2.573 | 0.01 |
|  | CDI | -0.304 | -1.85 | 0.064 |
|  | STAIC_trait | 0.32 | 2.532 | 0.011 |
|  | STAIC_status | 0.233 | 1.836 | 0.066 |
|  | age | -0.259 | -2.123 | 0.034 |
| SD of fixation locations | Congruency | 0.639 | 4.76 | 0 |
|  | Cue type | -0.387 | -2.879 | 0.004 |
|  | Group X Congruency | 0.382 | 1.826 | 0.068 |
|  | SOA(0.75) X Cue type | 0.294 | 1.55 | 0.121 |
|  | Congruency X Cue type | 0.509 | 2.681 | 0.007 |
|  | Group X SOA(0.75) X Cue type | -0.452 | -1.528 | 0.127 |
|  | CARS | 0.222 | 1.787 | 0.074 |
|  | CDI | -0.365 | -2.591 | 0.01 |
|  | Congruency | 0.342 | 3.152 | 0.002 |
|  | age | -0.264 | -2.524 | 0.012 |
| Rate of null data | Group | 0.478 | 1.474 | 0.14 |
|  | CDI | -0.28 | -2.023 | 0.043 |
|  | age | -0.158 | -1.535 | 0.125 |

Table 3. Linear mixed-effects model results for the social-cue condition (significant coefficients only).

| **DV** | **variable** | **coefficient** | **z** | **p** |
| --- | --- | --- | --- | --- |
| Rate of response to joint attention | Group | -0.454 | -1.448 | 0.148 |
|  | Congruency | -0.295 | -1.6 | 0.11 |
|  | CDI | -0.223 | -1.634 | 0.102 |
| Rate of peripheral vision | Group | 0.793 | 2.237 | 0.025 |
|  | SOA(0.5) | 0.312 | 1.737 | 0.082 |
|  | SOA(0.75) | 0.336 | 1.871 | 0.061 |
|  | Group X SOA(0.75) | -0.576 | -2.056 | 0.04 |
|  | CARS | -0.275 | -1.901 | 0.057 |
|  | CDI | 0.258 | 1.569 | 0.117 |
|  | STAIC_trait | -0.408 | -3.229 | 0.001 |
|  | age | 0.207 | 1.7 | 0.089 |
| Duration in each fixation during target detection | Group | 0.787 | 2.143 | 0.032 |
|  | Group X SOA(0.5) | -0.4 | -1.661 | 0.097 |

Table 2 Correlations between the KARS score and each feature selected for logistic regression.

| Category | Indicators | 1 | 2 | 3 | 4 | 5 | 6 | 7 | 8 | 9 | 10 |  |  |
| --- | --- | --- | --- | --- | --- | --- | --- | --- | --- | --- | --- | --- | --- |
| KARS score | 1. Inattention |  |  |  |  |  |  |  |  |  |  |  |  |
|  | 2. Hyperactivity | 0.84^***^ |  |  |  |  |  |  |  |  |  |  |  |
| Social | 3. Rate of JA  (0.25-s SOA/cong) | -0.3^**^ | -0.21 |  |  |  |  |  |  |  |  |  |  |
|  | 4. GCE in the number of saccades  (0.5-s SOA) | 0.35^**^ | 0.35^**^ | -0.2 |  |  |  |  |  |  |  |  |  |
|  | 5. JA score in the RT (0.5-s SOA) | -0.02 | -0.02 | -0.25 | 0.61^***^ |  |  |  |  |  |  |  |  |
|  | 6. Number of saccades  (0.25-s SOA/cong) | -0.23 | -0.11 | 0.14 | -0.0 | -0.04 |  |  |  |  |  |  |  |
|  | 7. Duration of each fixation point | 0.46^**^ | 0.36^**^ | -0.22 | -0.01 | 0.09 | -0.66^***^ |  |  |  |  |  |  |
| Nonsocial | 8. Number of saccades  (0.25-s SOA/incong) | -0.39^**^ | -0.35^**^ | 0.24 | -0.12 | 0.09 | 0.68^***^ | -0.49^***^ |  |  |  |  |  |
|  | 9. SD of fixation locations  (0.25-s SOA/incong) | -0.23 | -0.1 | 0.09 | 0.04 | -0.09 | 0.37^**^ | -0.45^**^ | 0.33^**^ |  |  |  |  |
|  | 10. null_rate  (0.5-s SOA/incong) | 0.36^**^ | 0.24 | -0.11 | 0.17 | 0.28 | -0.14 | 0.54^***^ | -0.05 | -0.17 |  |  |  |
|  | 11. Accuracy  (0.25-s SOA/incong) | -0.29 | -0.17 | 0.08 | -0.27 | -0.35^**^ | 0.13 | -0.17 | 0.06 | 0.47^**^ | -0.03 |  |  |
| Both | 12. Rate of nulls | 0.33^**^ | 0.24 | -0.07 | 0.18 | 0.26 | 0.04 | 0.33^**^ | 0.01 | -0.26 | 0.77^***^ | -0.17 |  |
|  | 13. Variance of RT | 0.27 | 0.32^**^ | -0.09 | 0.28 | 0.39^**^ | 0.36^**^ | 0.0 | 0.26 | -0.1 | 0.36^**^ | -0.5^***^ | 0.63^***^ |

JA: Joint attention; SOA: stimulus onset asynchrony; RT: reaction time; SD: standard deviation; cong/incong: congruent cue/incongruent cues.

^**^ indicates correlation is significant with *p* <.01

*^***^* indicates correlation is significant with *p* <.001
